# Modulation of Wnt Signaling Enhances Inner Ear Organoid Development in 3D Culture

**DOI:** 10.1371/journal.pone.0162508

**Published:** 2016-09-08

**Authors:** Rachel E. DeJonge, Xiao-Ping Liu, Christopher R. Deig, Stefan Heller, Karl R. Koehler, Eri Hashino

**Affiliations:** 1 Department of Otolaryngology-Head and Neck Surgery, Indiana University School of Medicine, Indianapolis, IN, 46202, United States of America; 2 Stark Neurosciences Research Institute, Indiana University School of Medicine, Indianapolis, IN, 46202, United States of America; 3 Department of Otolaryngology, F.M. Kirby Neurobiology Center Boston Children’s Hospital, and Harvard Medical School, Boston, MA, 02115, United States of America; 4 Department of Otolaryngology-Head and Neck Surgery, Stanford University, Palo Alto, CA, 94305, United States of America; Harvard University, UNITED STATES

## Abstract

Stem cell-derived inner ear sensory epithelia are a promising source of tissues for treating patients with hearing loss and dizziness. We recently demonstrated how to generate inner ear sensory epithelia, designated as inner ear organoids, from mouse embryonic stem cells (ESCs) in a self-organizing 3D culture. Here we improve the efficiency of this culture system by elucidating how Wnt signaling activity can drive the induction of otic tissue. We found that a carefully timed treatment with the potent Wnt agonist CHIR99021 promotes induction of otic vesicles—a process that was previously self-organized by unknown mechanisms. The resulting otic-like vesicles have a larger lumen size and contain a greater number of Pax8/Pax2-positive otic progenitor cells than organoids derived without the Wnt agonist. Additionally, these otic-like vesicles give rise to large inner ear organoids with hair cells whose morphological, biochemical and functional properties are indistinguishable from those of vestibular hair cells in the postnatal mouse inner ear. We conclude that Wnt signaling plays a similar role during inner ear organoid formation as it does during inner ear development in the embryo.

## Introduction

The sensory organs of the inner ear—the macula, cristae, and the Organ of Corti—develop from a symphony of complex spatiotemporal signaling mechanisms. These sensory organs allow for the detection of linear acceleration due to gravity, angular acceleration, and transduction of sound waves into nerve impulses. We previously reported that inner ear sensory epithelia could be generated from mouse pluripotent stem cells over a period of 14–20 days in 3D culture [1]. We first generated a non-neural epithelium and then induced an otic epibranchial pre-placodal epithelium by inhibiting bone morphogenetic protein (BMP) and activating fibroblast growth factor (FGF) signaling. A critical step in the latter process is the self-organized formation of otic vesicles within the cell aggregates. However, our inner ear induction protocol yields a variable quantity of organoids depending on various confounding factors, such as experimenters, laboratory conditions and mouse stem lines. To improve the utility of our inner ear organoid culture, we sought to identify an additional signaling modulator that could normalize or amplify the otic induction process.

Multiple signaling pathways including Wnt, FGF, Notch, BMP, retinoids, and sonic hedgehog (Shh) have been shown to play a critical role in both the establishment of the otic placode and further differentiation into epidermal structures, epibranchial placodes, and the entirety of the inner ear [2–8]. Of these signaling pathways, canonical Wnt signaling cascade appears to be of particular importance in the development of the otic placode *in vivo* [2, 9–20]. Moreover, inhibiting Wnt signaling with the potent tankyrase inhibitor XAV-939 at differentiation days 8–10 abolishes otic vesicle formation in our 3D culture [1], strongly suggesting that Wnt ligands synthesized in cells within aggregates are essential for otic placode induction in our organoid culture. Based on these previous studies, we hypothesized that augmenting canonical Wnt signaling in stem cell-derived aggregates by supplementing a Wnt agonist prior to otic placode formation could increase the number and the size of otic vesicles derived in 3D culture.

## Materials and Methods

### Embryonic stem cell culture

Three mouse embryonic stem cell (ESC) lines, R1 (generated by Dr. Andas Nagy’s laboratory, [21]), R1/E (purchased from ATCC, SCRC-1036), and *Atoh1/nGFP* (generated by Dr. Stefan Heller’s laboratory, [22]), as well as an *Oct4/eGFP* induced pluripotent stem cell (iPSC) line (generated by Dr. Stephane Viville’s laboratory, [23]) were used in the present study. These pluripotent stem cells were subjected to differentiation using the SFEBq protocol as described previously [1, 24], but with major modifications. On day 3 of the protocol, BMP4 (10 ng/mL) and SB-431542 (1 μM) were added to each well at 5X concentration in 25 μL of fresh media. On day 4, 4.25 or 4.5, FGF2 (25 ng/mL) and LDN-193189 (100 nM) were added to each well at 6X concentration in 25 μL of fresh media. The concentration of Matrigel was maintained at 2% (v/v) throughout days 1–8. On day 8 of differentiation, cell aggregates were washed twice with PBS and once with N2 media before being transferred to 96 well plates (Lipidure Coat, NOF) in 150 μL of N2 Medium containing 1% Matrigel (v/v) and in the presence or absence of CHIR99021 (Stemgent) at a concentration of 1 μM, 3 μM, or 10 μM. N2 Medium contained Advanced DMEM/F12, 1X N2 Supplement, 50 μg/mL Normocin (Invivogen) and 1 mM GlutaMax. After 48 hours the cell aggregates were transferred to 24 well plates (Lipidure Coat, NOF; 1–2 aggregates per well) suspended in 500 μL of N2 Medium. A half medium change was preformed every other day starting 48 hr after cell aggregates were transferred to a 24-well plate, on day 16 the volume of N2 media was increased to 1.0 mL.

### Signaling molecules and recombinant proteins

The following small molecules and recombinant proteins were used: recombinant human BMP4 (10 ng/mL; Stemgent), human FGF2 (25 ng/mL; Peprotech), SB-431542 (1 μM; Stemgent), LDN-193189 (1 μM; Stemgent) and CHIR99021 (1, 3, or 10 μM; Stemgent).

### Immunohistochemistry

Aggregates were fixed with 4% paraformaldehyde. The fixed specimens were cryoprotected with a serial treatment of 15% and 30% sucrose and embedded in tissue freezing medium. Frozen tissue blocks were sectioned into 10 or 12 μm cyrosections. For immunostaining, a 3% Goat or Horse Serum and 0.1% Triton-X100 solution was used for primary antibody incubation. An Alexa Fluor 488, 568, or 647 conjugated anti-mouse IgG or anti-goat IgG and an Alexa Fluor 568 or 647 conjugated anti-rabbit IgG (Invitrogen) were used as secondary antibodies. A DAPI counterstain was used to visualize cellular nuclei (ProLong Gold antifade reagent with DAPI, Life Technologies). Microscopy was performed on a Nikon TE2000 Inverted Microscope or an Olympus FV1000-MPE Confocal/Multiphoton Microscope.

The following antibodies were used: anti-E-cadherin (rabbit, Abcam; mouse, BD Biosciences); anti-Nanog (rabbit, Abcam); anti-Pax8 (rabbit, Abcam); anti-Pax2 (rabbit, Invitrogen; mouse, Abnova); anti-Sox2 (mouse, BD Biosciences); anti-myosin7a (rabbit, Proteus); anti-acetylated-α-Tubulin (mouse, Abcam); anti-TuJ1 (mouse, Covance); anti-Calbindin2 (mouse, Millipore); anti-Brn3A (mouse, Millipore); anti-Brn3C (mouse, Santa Cruz Biotechnology); anti-CtBP2 (mouse, BD Biosciences); anti-GFP (mouse, Santa Cruz); anti-GFP (rabbit, Abcam); anti-GFP (mouse, Life Technologies); anti-Espin (rabbit, Gift of Dr. James Bartles).

### Image Analysis

For all image quantification, 10–12 μm-thick serial sections were obtained from 6–8 aggregates per condition for a total of 24–32 aggregate slices per slide. One slide per experimental group was used for analysis, with data summed from at least 3 separate experiments. The luminal area, perimeter, and maximum diameter of Pax2/Ecad^+^ vesicles were quantified in day 10 and 14 aggregates using 40x magnified images and NIH ImageJ software. The 50 μm scale bar was calibrated to pixel distance to obtain a measure of area in μm^2^. To analyze area, images were converted to 8-bit, threshold was adjusted, the vesicle was selected using the wand tracing tool, and the area was measured. In slices with multiple vesicles, outputs were averaged to obtain a measure of the average luminal area. For day 14 and 21 aggregates, Pax2/Ecad^+^ and Myo7a/Sox2^+^ vesicles were counted respectively. The number of vesicles were counted per aggregate slice to compare the average number of vesicles produced between groups.

### Electrophysiology

CHIR-treated organoids were transported by overnight delivery chilled in Hibernate A medium. Organoids were dissected open and stabilized under nylon strands with the bundles facing up. Hair cells were visualized with Nomarski optics at 63x on a Zeiss Axioskop FS inverted microscope, and recorded from in whole-cell patch clamp mode using an Axopatch 200B amplifier (Molecular Devices, Sunnyvale, CA). The external solution contained (in mM): 137 NaCl, 5.8 KCl, 0.7 NaH_2_PO_4_, 10 HEPES, 1.3 CaCl_2_, 0.9 MgCl_2_, 5.6 glucose, vitamins and essential amino acids (Invitrogen, Carlsbad, CA), adjusted to pH 7.4, ~310 mmol/kg. Electrodes were filled with a solution containing 135 KCl, 5 HEPES, 5 EGTA, 2.5 MgCl_2_, 2.5 K_2_‐ATP, 0.1 CaCl_2_, adjusted to pH 7.4, ~285 mmol/kg. Voltages were corrected for a 5 mV junction potential. For mechanotransduction, the kinocilium was coupled by gentle suction into a glass pipette attached to a piezoelectric bimorph controlled by a piezo driver (MDT694, Thor Labs, Newton, NJ) filtered at 1 kHz.

### Statistical Analysis

Statistical significance was determined using a Student’s t-test for comparison of two groups or a one-way ANOVA followed by Tukey’s *post-hoc* test for multiple comparisons, unless stated otherwise. All data were analyzed using Prism 6 (GraphPad), IBM SPSS Statistics 22, or Microsoft Excel software.

## Results

**Fig 1:**
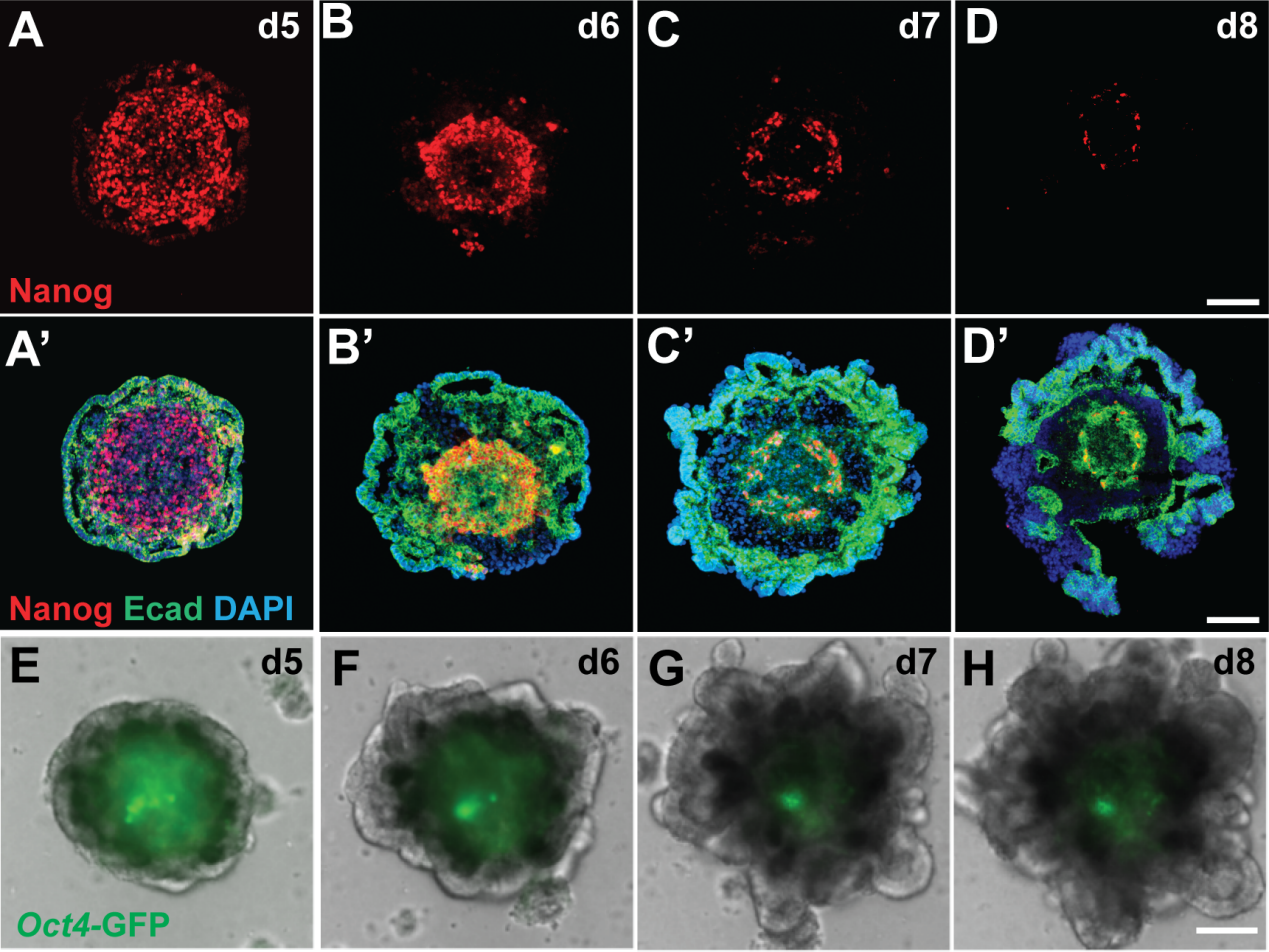
Spatio-temporal changes in expression of pluripotency markers and self-organization of stem cell-derived aggregates in 3D culture. (A-D) Expression of the pluripotency marker Nanog decreases as vesicles mature between differentiation days 5–8. (A’-D’) Progression of outer epithelium ruffling indicating self-organization. (E-H) Oct4-GFP expressing pluripotent stem cells decrease in number and are confined to the aggregate core by differentiation day 8. Scale bars, 100 μm (D, D’, H).

To evaluate the reproducibility of our inner ear induction protocol [1, 24], we applied it to several different mouse pluripotent stem cell lines, including wild-type R1 [21], wild-type R1/E, *Atoh1/nGFP* [22] ESCs and *Oct4/eGFP* iPSCs [23]. Aggregates from all stem cell lines grew at a similar rate and generated outer-epithelia that thickened following treatment with BMP4 and the TGFβ inhibitor SB-431542 at day 3 followed by FGF2 and the BMP inhibitor LDN-193189 at day 4–4.5 (Fig 1A’–1D’ and 1E–1H). We found that the optimal timing for the FGF2 and LDN-193189 treatment differs among different stem cell lines with the *Atoh1/nGFP* line at day 4, R1 and R1/E lines at day 4.25 and the *Oct4/eGFP* line at day 4.5. Nanog immunofluorescence and *Oct4* reporter expression consistently demonstrate that pluripotent stem cells gradually decrease in number and become confined to the inner core of the aggregates by day 8 after the start of differentiation (Fig 1A–1D and 1E–1H). Starting at day 7, we started to see ruffling of the outer epithelial region (Fig 1A’–1D’ and 1E–1H) indicating self-organization of the cell aggregates.

Beginning on day 8 of the protocol, the outer-epithelium becomes enveloped by migrating mesodermal and neural cells from the aggregate core. Otic vesicles then evaginate from the epithelium into this migrating mass of cells. Of note, we have never observed Pax2 protein expression using immunohistochemistry until after this cell rearrangement has begun (i.e. day 9 and beyond). Thus, we reasoned that this is a critical point in determining the outcome of otic induction and endogenous factors or mechanical cues from the migratory cell population likely drive otic morphogenesis. We previously showed that inhibiting Wnt signaling during this time period abolished otic vesicle formation [1]; thus, we wondered whether addition of a Wnt agonist could enhance otic vesicle formation.

**Fig 2:**
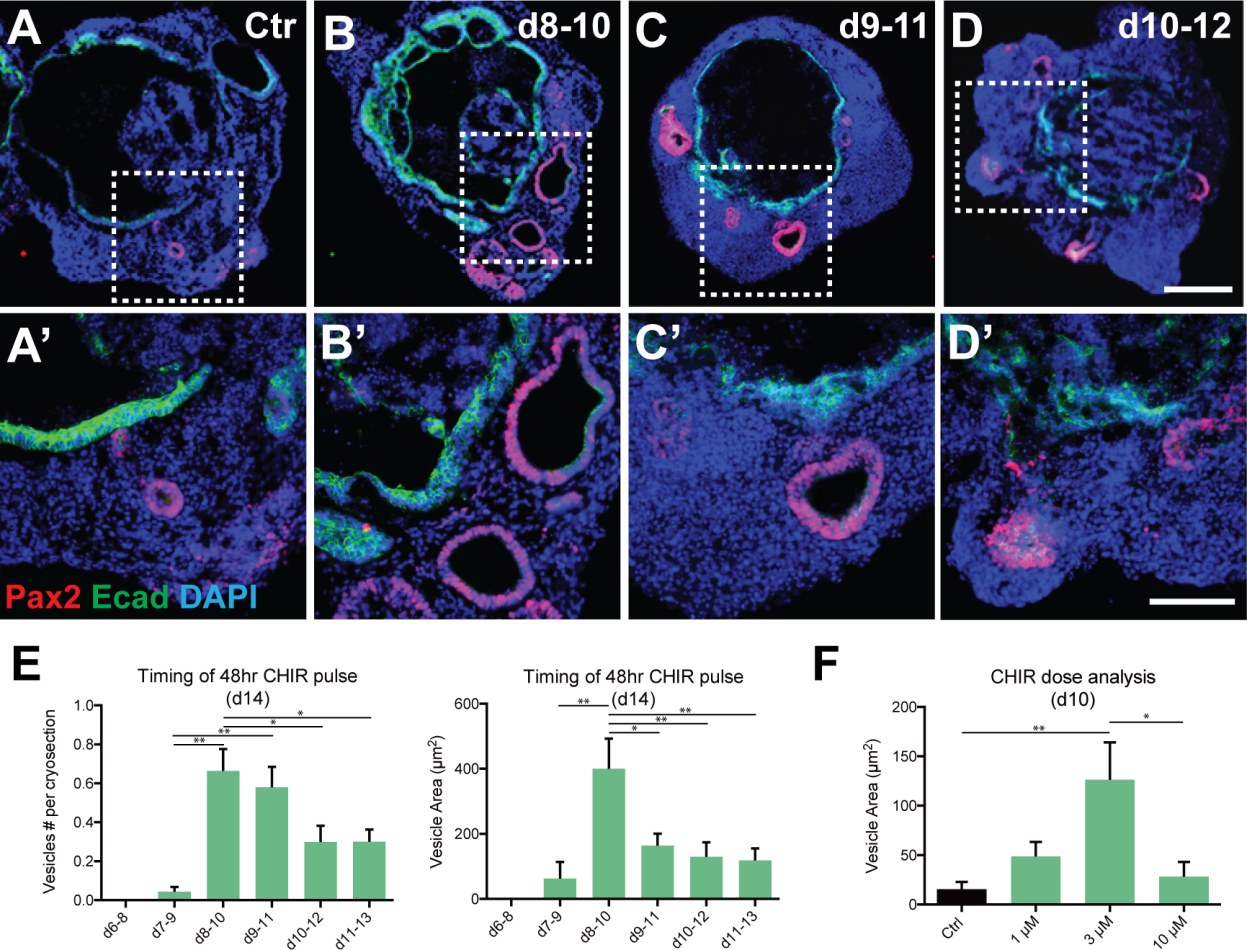
CHIR treatment exerts time- and dose-dependent effects on otic vesicle derivation in 3D culture. (A-D’) Pax2^+^/Ecad^+^ otic vesicles in R1 ESC-derived aggregates treated with CHIR for 48hrs starting at day 8 (B, B’), 9 (C, C’) or 10 (D, D’) along with untreated controls (A, A’). (E) Comparison of the number and the area size of Pax2^+^/Ecad^+^ vesicles per aggregate among experimental groups treated with CHIR at different starting times (*P<0.05, **P<0.001; mean ± s.e.m.; n = 42–80 per group). (F) Comparison of the area size of Pax2^+^/Ecad^+^ vesicles per aggregate among experimental groups treated with CHIR at different concentrations. (*P<0.05, **P<0.001; mean ± s.e.m.; n = 52–66 per group). Scale bars, 100 μm (D), 50 μm (D’).

To determine whether augmentation of Wnt signaling could enhance otic differentiation [25], we treated aggregates of R1 ESCs with CHIR99021 (CHIR), a GSK3ß inhibitor and potent Wnt agonist, for 48 hrs starting at day 6, 7, 8, 9, 10 and 11 of differentiation. All aggregates were collected on day 12 and the mean Pax2/E-cadherin (Ecad)^+^ luminal area and the number of vesicles were compared among different treatment groups (Fig 2A–2E). Day 8 CHIR treatment resulted in a higher mean vesicle production (0.66 ± 0.11) than CHIR addition on day 7 (0.04 ± 0.02), day 10 (0.30 ± 0.08), or day 11 (0.30 ± 0.06). Day 9 CHIR treatment also resulted in favorable vesicle production with a larger mean number produced (0.58 ± 0.37) than day 7 CHIR treatment (0.04 ± 0.02). Day 14 analysis of luminal area confirmed day 8 as the optimal starting time for CHIR addition with a larger mean luminal area (400.37±92.63 μm^2^) than treatment on day 7 (62.90 ± 51.01 μm^2^), day 9 (163 ± 37.21 μm^2^), day 10 (129.82 ± 44.66 μm^2^), or day 11 (118.27 ± 36.75 μm^2^). No vesicles were produced with day 6 CHIR treatment, indicating that early Wnt activation is detrimental for otic vesicle formation. Day 6 aggregates also differed noticeably from other treatment groups with a much larger aggregate size made up of bulbous projections and loose cell clumps. Pax8 and Ecad expression indicates that the pre-placodal ectoderm is forming between days 6–8 [1]; addition of CHIR before the epithelium can thicken, ruffle and form ovoid vesicles seems to inhibit further determination of otic fate including the differentiation of hair cells.

CHIR dose-response was compared between 0, 1, 3, and 10 μM CHIR, with a 48- hour treatment beginning on day 8 (Fig 2F). Quantification of culture day 10 Pax2/Ecad^+^ vesicles showed increased mean luminal area in aggregates treated with 3 μM CHIR (126.21 ± 37.93 μm^2^) compared to those with no CHIR (15.27 ± 7.41 μm^2^) and 10 μM CHIR (28.21 ± 14.85 μm^2^). A non-significant trend towards increased mean luminal area was observed with 3 μM CHIR compared to 1 μM CHIR (48.57 ± 14.90 μm^2^). On day 21, the mean number of Myo7a/Sox2^+^ vesicles was higher in aggregates treated with 3 μM CHIR (1.28 ± 0.17) compared to no CHIR (0.54 ± 0.13) and 10 μM CHIR (0.31 ± 0.07). 1 μM CHIR treatment resulted in a higher mean vesicle number (1.28 ± 0.17) than 10 μM CHIR (0.31 ± 0.07) but not higher than aggregates without CHIR treatment (0.54 ± 0.13). Based on dose response data, we concluded that the 3 μM CHIR dose was optimal for use in subsequent experiments.

**Fig 3:**
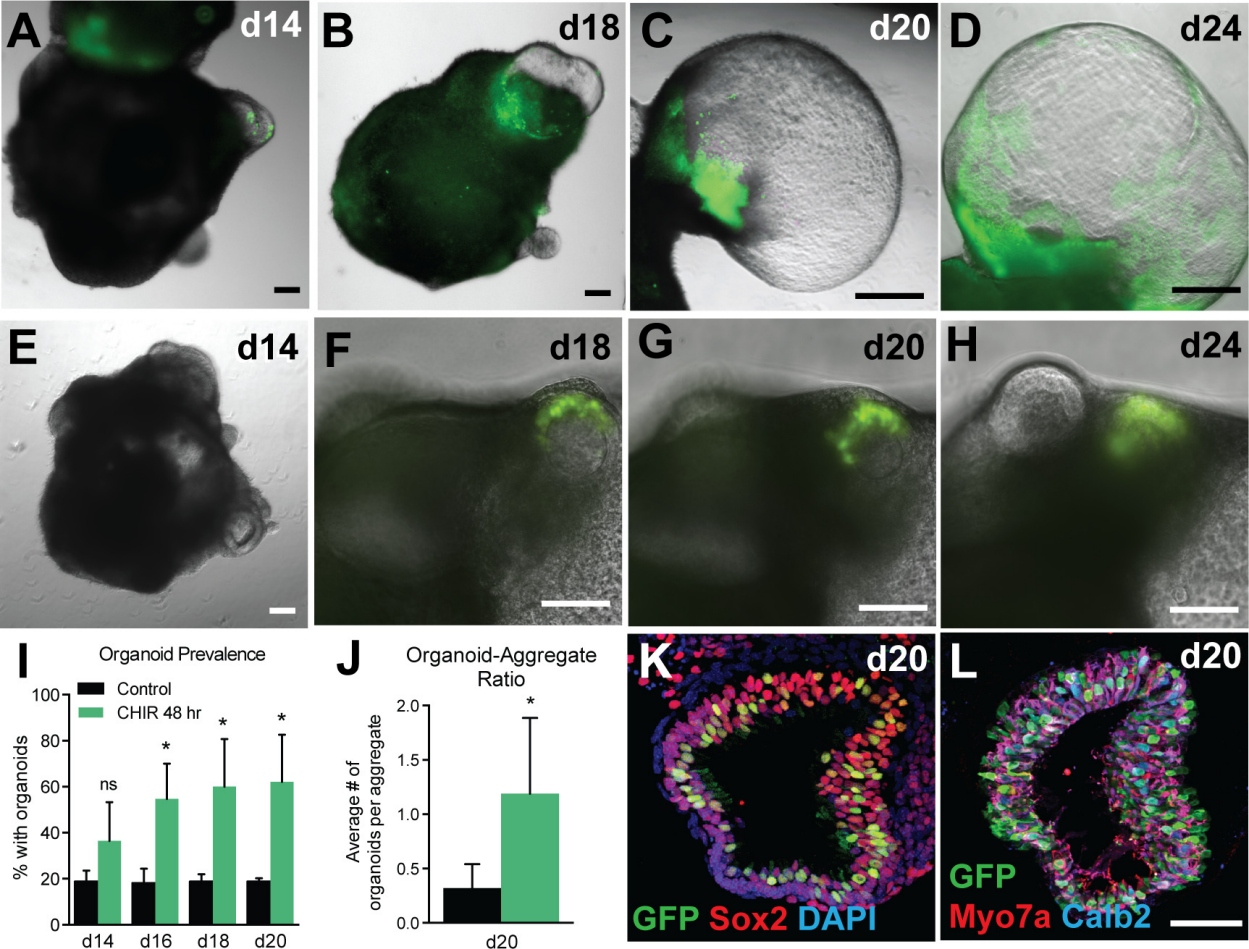
CHIR treatment increases the prevalence and size of *Atoh1/nGFP*–positive inner ear organoids in 3D culture. (A-D’) *Atoh1/nGFP*–positive sensory patches in a protruding vesicle become larger with time in aggregates treated with CHIR. (E-H) There is no noticeable temporal change in the size of *Atoh1/nGFP*–positive sensory patches in untreated control aggregates. (I) Comparison of the percentage of aggregates containing at least one *Atoh1/nGFP*–positive vesicles between CHIR-treated aggregates and untreated controls (*P < 0.05; n = 14 per group). (J) The average number of *Atoh1/nGFP*–positive organoids per aggregate in CHIR-treated aggregates and untreated controls (*P < 0.05; n = 14 per group). (K-L) Immunoflorescence showing *Atoh1/nGFP*-positive cells express Sox2 and Myo7A. Scale bars, 100 μm (D), 10 μm (D’, H).

To evaluate the effects of CHIR on hair cell induction, *Atoh1/nGFP* ESCs were subjected to the otic induction in the presence or absence of CHIR and temporal changes in *Atoh1/nGFP* expression were monitored daily starting at day 14 of differentiation. In CHIR treated aggregates, GFP expression in protruding vesicles was observed by day 14, and the area with GFP-positive cells as well as the size of the vesicles become larger with time (Fig 3A–3D). In contrast, no notable changes in the size of GFP-positive vesicles were observed in untreated aggregates between days 18 and 24 (Fig 3E–3H). Quantitative analysis revealed that the percentage of aggregates containing at least one GFP-positive vesicle in the CHIR treated group was significantly higher than that in the untreated control group between days 16 and 20 (Fig 3I). Moreover, the average number of GFP-positive vesicles per aggregate in the CHIR treated group was significantly higher than that in the untreated control group (Fig 3J). The majority of GFP-positive cells in those vesicles express Sox2 and Myosin7a (Myo7a; Fig 3K and 3L), indicating that vesicles containing GFP-positive cells contain hair cells and not other Atoh1-expressing cells, such as hindbrain neural progenitors.

**Fig 4:**
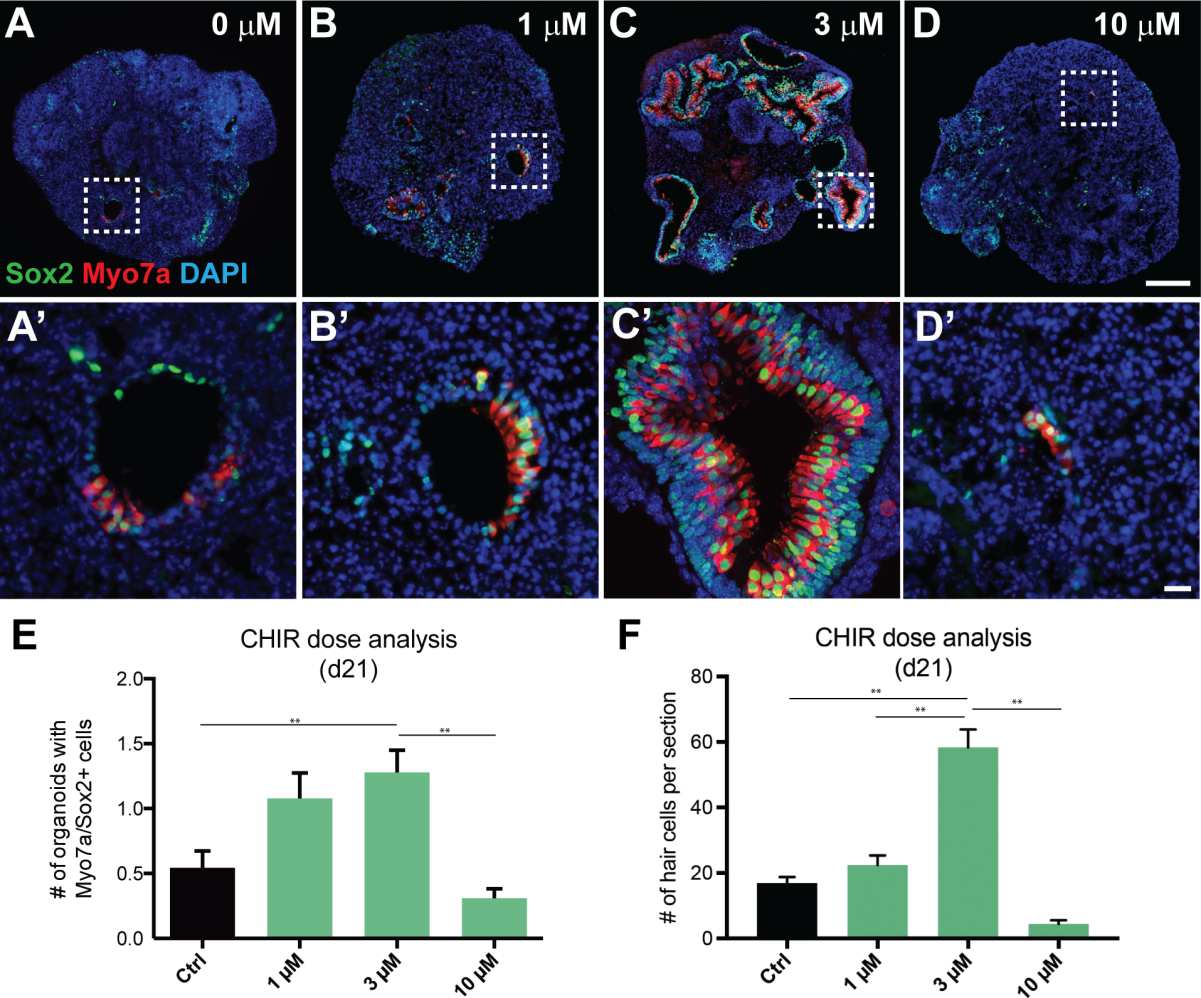
CHIR treatment has dose-dependent effects on the number of vesicles containing Myo7a^+^/Sox2^+^ cells. (A-D’) Representative images show Myo7a/Sox2 expression in day 21 aggregate sensory epithelia that received 0 μM (A, A’), 1 μM (B, B’), 3 μM (C, C’), and 10 μM (D, D’) CHIR between days 8 and 10. (E) The average number of vesicles containing Myo7a^+^/Sox2^+^ cells per aggregate as a function of the CHIR concentration (**P < 0.001; mean ± s.e.m.; n = 55–68 per group). (F) The largest number of Myo7a^+^/Sox2^+^ cells per section as a function of the CHIR concentration (**P < 0.001; mean ± s.e.m.; n = 10 per group). Scale bars, 100 μm (D), 10 μm (D’, H).

To test if CHIR treatment has long-lasting effects and enhances derivation of Myo7a/Sox2-positive cells, we grew ESC-derived aggregates in the presence or absence CHIR for 48 hrs from day 8 to day 10 and maintained these aggregates for an additional 11 days. We consistently observed dose-dependent effects of CHIR on the number of Myo7a/Sox2-positive cells with 3 μM CHIR yielding the greatest effects on the number of Myo7a/Sox2-positive cells among the other concentrations (0, 1 and 10 μM; Fig 4). These results confirm that treating ESC-derived aggregates with 3 μM for 48 hrs between days 8 and 10 after the start of differentiation appears to represent the optimal condition for generating the largest number of Pax2/Ecad-positive otic progenitors that subsequently give rise to Myo7a/Sox2-positive cells.

**Fig 5:**
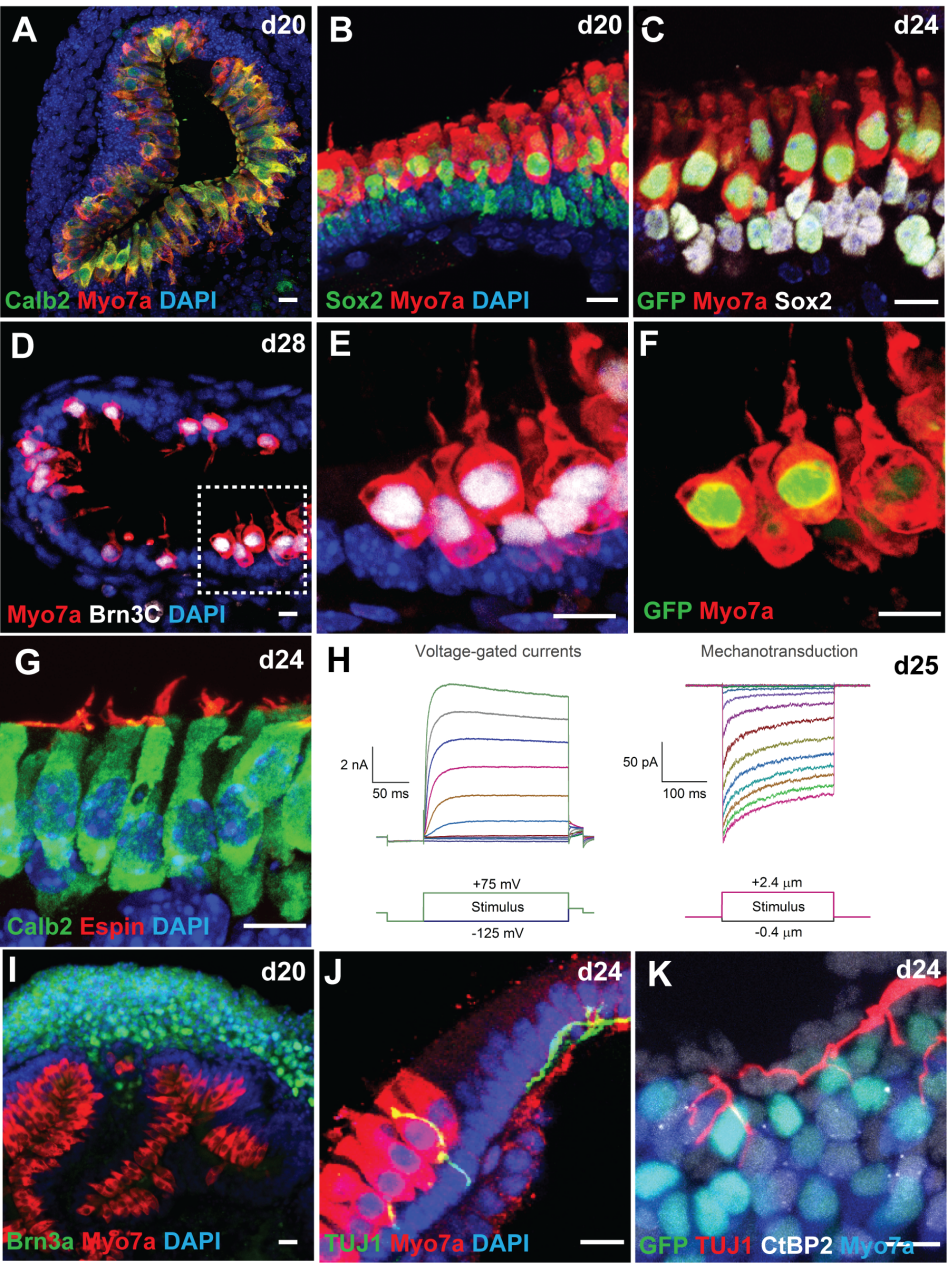
CHIR-treated aggregates give rise to inner ear organoids harboring mechanosensitive hair cells. (A-B) Co-localization of two hair cell markers Calb2 and Myo7a (A) or Sox2 and Myo7a (B) in cells lining the luminal surface of a vesicle. (C) *Atoh1/nGFP*^*+*^ cells also express Myo7a and Sox2. (D-F) Cells expressing Brn3C, Myo7a, and *Atoh1/nGFP* exhibit flask-like morphology with a hair bundle on their apical surface characteristic of vestibular hair cells. Some Myo7a^+^ cells in day 28 samples have faint *Atoh1/nGFP* expression (arrows), suggesting that these cells are more mature hair cells than cells expressing strong *Atoh1/nGFP* expression. (G) The hair bundle marker Espin was observed on the apical surface of Calb2^+^ hair cells. (H) Representative voltage-gated currents and mechanosensitive currents recorded from day 25 *Atoh1/nGFP*^*+*^ cells in response to voltage injections and hair bundle deflections, respectively. (I) A cluster of Brn3A^+^ neuronal cell bodies were located near Myo7a^+^ hair cells. (J) A TUJ1^+^ neural processes extend and contact Myo7a^+^ hair cells. (K) The ribbon synapse marker CtBP2 was associated with Myo7a^+^ cells and TUJ1^+^ processes. Scale bars, 10 μm (A-G, I-K).

To test whether CHIR-treated ESC-derived aggregates give rise to sensory hair cells with morphological, biochemical and functional properties of native hair cells, we first performed immunofluorescence analysis of day 20–28 samples for multiple hair cell markers, including Myo7a, Calbindin2 (Calb2) and Brn3C. We found that the majority of *Atoh1/nGFP* positive cells express these marker proteins and exhibit cylindrical or flask-like morphology characteristic of Type I and II vestibular hair cells, respectively (Fig 5A–5F). In addition, Espin-positive hair bundles were observed on the apical surface of Calb2-positive cells (Fig 5G). Whole-cell patch-clamp recordings from *Atoh1/nGFP* positive cells with a hair bundle revealed that these hair cells generate mechanotransduction currents and voltage-gated potassium currents similar to those typically observed from native vestibular hair cells in post-natal mouse inner ear (Fig 5H). Upon deflecting the hair bundles, we observed inward currents characteristic of transduction currents, exhibiting adaptation and displacement sensitivity similar to those of postnatal vestibular hair cells. Derived hair cells had negative resting potentials and exhibited voltage-gated currents typical of normal hair cells, such as the presence of large outward K^+^ currents, and fast inward rectifying K^+^ currents.

In addition to normal development of the sensory epithelia, we observed a large cluster of Brn3C and HuC/D positive neuronal cell bodies in the vicinity of Myo7a-positive epithelia (Fig 5I). Three-dimensional reconstruction of a day-25 aggregate stained for Myo7a and TUJ1 reveals that some of the neurons appear to extend their processes towards Myo7a-positive cells (Fig 5J, S1 Movie). Moreover, we observed CtBP2-positive puncta, a marker for ribbon synapses, associated with both Myo7a-positive cells and TUJ1-positive processes (Fig 5K). These results suggest that vestibulocochlear-like ganglia containing sensory neurons are being formed adjacent to the sensory epithelia and extend their processes and make synaptic connections with derived hair cells in the organoid.

## Discussion

We have found that our previously reported inner ear induction protocol yields a variable quantity of organoids across mouse ESC lines, experimenters and laboratories, with some cases producing organoids in <1% of the aggregates (Jonathan Gale and Kimiko Kato, personal communication). To improve the utility of our inner ear organoid culture, we sought in this study to identify additional signaling modulator(s) that could normalize or amplify the otic induction process.

The otic placode undergoes multiple invaginations to form the otic pit before becoming the otic vesicle under the influence of activated Wnt signaling emanating from the dorsal hindbrain. The otic vesicle further develops into both the sensory and non-sensory portions of the inner ear [13]. The portion of the otic epibranchial pre-placodal epithelium nearest the neural tube receives higher levels of Wnt signaling, which drives the cells towards an otic fate. The more lateral cells that receive less Wnt and more FGF signaling form epidermal and epibranchial structures [6]. The role for Wnt signaling in this process has been shown in multiple studies: XAV-939, a potent inhibitor of the Wnt cascade, reduces the number of otic vesicles in a 3D *in vitro* mouse model of inner ear sensory epithelia [1, 24]; β-catenin mouse knockouts lack sensory hair cells in the inner ear [18]; inhibition of Wnt signaling reduces otic vesicle size [6, 10, 26–28]; treatment with the Wnt agonist BIO during somitogenesis results in enlarged otic vesicles and increased Pax2a expression in zebrafish embryos [29]; a Wnt antagonist, Dkk1, blocks expression of the inner ear marker, Soho1, without blocking expression of Pax2 [10]. In multiple organisms including *Xenopus*, zebrafish, chick, and mouse, the combination of Wnt and FGF signaling activates Pax8 expression and together specify the otic placode, while knockdown reduces expression of otic markers or decreases the size of otic vesicles [12, 14, 17, 26, 28, 30, 31]. However, because otic placode development has been observed without Wnt8 expression in zebrafish, Wnt signaling may function to direct placodal fate over epidermal in Pax2^+^ precursor cells [6, 27]. In addition, Wnt restricts neurogenesis in the otic vesicle via modulation of the genes Tbx1, Eya1, and Six1, which may allow for expanded sensory domains within the inner ear [2, 32]. Single-cell expression analysis has revealed that in order for neuroblasts to develop into epibranchial placodes and their associated neural ganglia, they must lose their responsiveness to Wnt during development [33].

**Fig 6:**
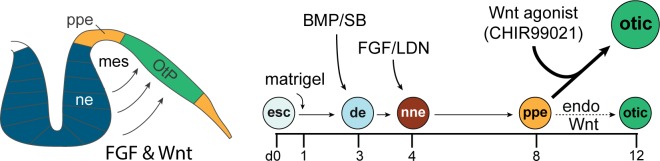
Schematic summary of the proposed role of Wnt signaling in inner ear organoid formation in 3D culture. Augmentation of canonical Wnt signaling by CHIR99021, a potent Wnt agonist, upon preplacodal formation in stem cell-derived aggregates promotes otic vesicle formation at the expense of other preplacodal derivatives, resulting in the formation of a larger number of hair cells. ne, neural ectoderm. ppe, pre-placodal epithelium. OtP, otic placode. mes, mesoderm. esc, embryonic stem cell. de, definitive ectoderm. nne, non-neural ectoderm.

Consistent with the well-established role for canonical Wnt signaling in otic placode induction *in vivo*, we have demonstrated in the present study that precisely timed augmentation of canonical Wnt signaling promotes induction of Pax2/Pax8-positive otic progenitor cells in mouse ESC-derived inner ear organoids *in vitro* (Fig 6). CHIR acts as a pharmacological activator of Wnt signaling by inhibiting GSK3 and promoting nuclear accumulation of beta-catenin, which leads to activation of Wnt-target genes. Treating ESC-derived aggregates with CHIR on or after the start of Pax8 expression (day 8), but before the start of Pax2 expression (day 10) significantly increases the number of Pax2-positive cells (Fig 2), which results in a larger number of otic-like vesicles (Fig 3) and Myo7a-positive cells (Fig 4). CHIR appears to have little effect on ESC-derived aggregates when it is applied to the aggregates before Pax8 expression (Fig 2E). This is consistent with inner ear development *in vivo* where Wnt signaling appears to be necessary for otic placode induction *after* specification of the broader Pax8+ otic-epibranchial progenitor domain [15, 34]. As in inner ear development, in which Wnt ligands are thought to diffuse from the neural tube between embryonic days 7.5–8.5, our model appears to have a temporal window for Wnt activation in which otic induction is optimal [6, 35–38]. In addition, the effects of CHIR was dose-dependent with 3 μM CHIR to be most efficacious (Figs 2F and 4A–4F). By contrast, CHIR at 10 μM seemed to have toxic and undesirable effects on the overall viability of cell aggregates.

CHIR-treated aggregates give rise to large vesicles, some of which protrude onto the surface to become cyst-like structures. The luminal surface of these cysts was populated by cells expressing multiple-hair cell markers, including Atoh1, Myo7a, Calb2 and Brn3C, with Espin-positive hair bundles on their apical surface (Fig 5A–5G). Single cell patch-clamp recordings from these hair bundle-bearing cells revealed that they elicit mechano-transduction currents and voltage-gated potassium currents, characteristic of post-natal vestibular hair cells in the mouse inner ear (Fig 5H). In a recent study, we conducted an in-depth analysis of electrophysiology properties of hair cells and neurons in organoids and found that stem cell-derived hair cells progress through a similar dynamic developmental pattern of ion channel expression with native mouse vestibular hair cells [39]. These results indicate that timed application of CHIR not only enhances otic vesicle formation, but also increases the number of functional hair cells derived from mouse ESCs. Moreover, the treatment appears to be effective for different ESC lines.

In summary, we have devised a modified protocol to generate a larger number of otic progenitors that are competent to give rise to functional sensory hair cells within 25 days *in vitro*. The protocol could be further optimized to yield even larger numbers of hair cells by activating or suppressing other signaling pathways. This improved culture system will be more apt for chemical screening or toxicity testing than our original protocol due to the robustness and consistency of hair cell derivation.

## Supporting Information

S1 Movie Confocal 3D reconstruction of a day 25 ESC-derived organoid containing Myo7a^+^ hair cells (red), TUJ1^+^ neurons (green) and DAPI^+^ cellular nuclei (blue).
(MOV)
